# Supplementary material for: Tracking the Epidemiologic Shifts in Hepatitis A Sero-Prevalence Using Age Stratification: A Cross-Sectional Study at Jordan University Hospital
Source: Pathogens. 2021 Aug 26;10(9):1081. doi: 10.3390/pathogens10091081 (PMC8468970; doi:10.3390/pathogens10091081)
Supplement: Supplementary file 1 [file pathogens-10-01081-s001.zip › pathogens-1335583-supplementary.pdf]

Supplementary material

Study Subject Consent Form and Questionnaire

The University of Jordan Hospital

Subject Information and Consent Form

To whom it might concern

I am the undersigned ……………………………, after explanation by my doctor, I agree willingly to participate and enter the research entitled:

“Tracking the epidemiologic shifts in hepatitis A sero-prevalence using age stratification: A cross-sectional study at Jordan University Hospital”

I also agree to give the necessary samples to the research team and to adhere to the dates of follow-up visits. I authorize my doctor to give the necessary information about me to the study team for the necessary scientific uses, provided that my identity will remain unidentified and that my participation in the study does not affect the quality of the treatment I receive.

Name of the participant: …………………………….

Signature of the participant: …………………………….

Age/Date of Birth: …………………………….

Date: …………………………….

Witness: …………………………….

ID No.: …………………………….

Thank you very much for agreeing to participate in this Research. The information provided by you in this questionnaire will be used for research purposes. It will not be used in a manner which would allow identification of your individual responses.

Age: ………………….

Address: ………………….

Gender:

- ☐ Male
- ☐ Female

Nationality:

- ☐ Jordanian
- ☐ Non-Jordanian

Monthly Income:

- ☐ Less than 500 JD
- ☐ 500-1000 JD
- ☐ More than 1000 JD

Educational Level:

- ☐ High school or less
- ☐ Undergraduate
- ☐ Postgraduate

Drinking Water Source:

- ☐ Mineral water (bottled water)
- ☐ Water filtered at home (if positive, please indicate the filter age: ………………….)
- ☐ Water filtered from a local company
- ☐ Others, please indicate………………………

Number of Household Inhabitants:

☐ ……………………

Based on your weekly consumption of fast food (including falafel and shawarma), and on a scale from 1 to 10, how do you rate yourself?

☐ 1 (Lack of consumption); ☐ 2; ☐ 3; ☐ 4; ☐ 5; ☐ 6; ☐ 7; ☐ 8; ☐ 9; ☐ 10 (daily consumption)

Thank You
